# Intra-operative rapid diagnostic method based on CK19 mRNA expression for the detection of lymph node metastases in breast cancer

**DOI:** 10.1002/ijc.23451

**Published:** 2008-03-06

**Authors:** Mike Visser, Mehdi Jiwa, Anja Horstman, Antoinette ATP Brink, Rene P Pol, Paul van Diest, Peter JF Snijders, Chris JLM Meijer

**Affiliations:** 1 Department of Pathology, VU Medical Center AmsterdamThe Netherlands; 2 Department of Pathology, Medical Center AlkmaarThe Netherlands; 3 Department of Pathology, University Medical CenterUtrecht, The Netherlands

**Keywords:** OSNA, intra-operative, lymph node metastases, breast cancer, cytokeratin 19

## Abstract

Staging by sentinel node (SN) biopsy is the standard procedure for clinically node-negative breast cancer patients. Intra-operative analysis of the SN allows immediate axillary lymph node (ALN) dissection in SN positive patients, but a quick, reliable and reproducible method is lacking. We tested the suitability of a quantitative cytokeratin 19 (CK19) mRNA one step nucleic acid amplification (OSNA#) technique (OSNA-CK19) for intra-operative SN analysis. OSNA-CK19 involves a short manual sample preparation step and subsequent fully automated amplification of CK19 mRNA based on reverse transcription loop-mediated isothermal amplification, with results available within 30–40 min. OSNA-CK19 was compared to histological staining (Hematoxylin&Eosin and CAM5.2 and CK19 immunostaining) of 346 frozen ALNs from 32 breast cancer patients, using half of the lymph node for each method. 267 samples were negative and 61 positive by both methods. Three samples were histology positive and OSNA-CK19 negative. Fifteen samples were histology negative and OSNA-CK19 positive, 11 of which had copy numbers close to the cut-off level of OSNA-CK19. Seven of these 15 samples were RT-PCR positive for epithelial markers and/or showed CK19 protein expression by Western blot suggesting the presence of tumor deposits in the lymph node part investigated by OSNA-CK19. Concordance with histology was 94.8%, and 96.8% after exclusion of the latter 7 discordant cases. Sensitivity was 95.3% and specificity was 94.7% before and 97.1% after discordant case investigation. Our results indicate that OSNA-CK19 can potentially be useful in an intra-operative clinical setting to detect SN tumor involvement in breast cancer patients.

The negative status of the sentinel lymph node (SN) in clinically lymph node-negative breast cancer patients is highly predictive for the negative status of the remaining axillary lymph nodes (ALN), and hence SN mapping has become a routine procedure for surgical staging of breast cancer patients.^1^–^4^ Rapid techniques, which are used to identify tumor deposits such as touch imprint preparations and cytological smears lack sufficient sensitivity.^5^,^6^ Consecutive frozen sections can also be done intra-operatively but also show a suboptimal sensitivity of in between 70 and 90%.^7^,^8^ Therefore, in many laboratories SN analysis is usually performed postoperatively by staining consecutive step formalin-fixed tissue sections at 3–5 levels, depending on the size of the lymph node, with Hematoxylin and Eosin (H&E) and a pan cytokeratin marker.^9^,^10^ However, this thorough assessment of formalin-fixed tissue is not applicable for intra-operative testing.^11^ As a consequence, the patient needs to be subjected to ALN dissection (ALND) in a second surgery session, if the postoperative histological examination of the SN turns out to be positive. Therefore, there is a need for a rapid, highly sensitive and specific method, which can be used for intra-operative assessment of the SN status.

Molecular approaches such as real-time PCR have been applied for the detection of tumor deposits in lymph nodes of breast cancer patients and indicated higher sensitivity than histological investigations.^12^–^14^ Results obtained with RT-PCR correlated with traditional predictors of prognosis^13^

CK19 mRNA is a suitable marker for identifying breast cancer deposits in lymph nodes because virtually all breast cancers express this cytoskeleton protein.^15^ Recently, a new semi-automated molecular method for rapid intra-operative diagnosis of lymph node metastases in breast cancer patients has been developed using One step nucleic acid amplification (OSNA). The OSNA-CK19 assay (Sysmex, Kobe, Japan) is based on homogenisation of lymph node samples followed by real-time amplification and quantitation of cytokeratin 19 (CK19) mRNA directly from the lysate, with results available within 30 min for one SN and 40 min for 4 SNs. The quantitative molecular result is related to the size of the metastases.

In recent studies performed in Japan OSNA-CK19 has been found to be a potentially valuable intra-operative method for the detection of lymph node metastases in patients with gastric,^16^ colorectal,^17^ and breast cancer.^18^ To find out whether this method has also potential to be a good intra-operative alternative for a more extensive postoperative histological work-up of SNs that is common in many European settings, we tested the performance of the OSNA-CK19 method in comparison with the standard histological method (staining step sections with H&E and pan cytokeratin staining) in 346 ALN from 32 Dutch breast cancer patients undergoing ALND.

## Material and Methods

### Patients and source of lymph nodes

Three hundred and forty six fresh lymph nodes were obtained from ALND specimens of 32 breast cancer patients undergoing axillary dissection in the Medical Centre Alkmaar, Alkmaar, the Netherlands, and the VU University medical center in Amsterdam from June 2005 to July 2006. These included 14 patients that underwent a SN procedure of which only non-SNs were analyzed in our study. The remaining 18 patients did not undergo a SN procedure. Patients receiving neoadjuvant therapy were excluded from the study. Patient characteristics are shown in Table I. Patients were staged according to the TNM classification.^19^

**Table I:** Clinicopathological Characteristics of Patients

	Number of patients
Stage	
0	0
I A/B	8
II A/B	15
III A/B/C	7
IV	2
Nodal status	
pN0	14
pN1	10
pN2	6
pN3	2
Histopathological type	
Invasive ductal carcinoma	30
Invasive lobular carcinoma	2

### Study design

**Figure 1:**
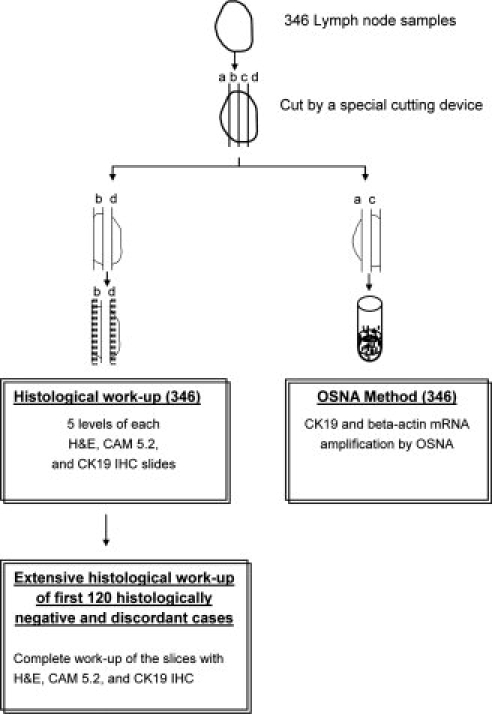
Study design: Histology *versus* OSNA. 346 lymph node samples were cut into 4 pieces. Slices “a” and “c” were subjected to the OSNA method, slices “b” and “c” to histological work-up consisting of 5 levels of H&E, CAM5.2 and CK19 staining. In 120 histologically negative lymph node samples, as determined by the 5 level method, the remainder of the block was completely cut into further levels to assess specificity. 18 samples with differing results as obtained by the 2 methods (discordant cases) were also cut into further levels.

Lymph node samples were cut in 4 equal slices (a, b, c, d) with a special cutting device.^18^ Two of these slices (a&c) were snap-frozen in liquid nitrogen and stored at −80°C until OSNA analysis was performed. The remaining 2 slices (b&d) were fixed in 4% buffered formaldehyde and embedded in a single paraffin block for histological examination at 5 levels since this was the standard in-house method for sentinel node investigation in both breast cancer and melanoma patients (Fig. 1).^20^

In 346 lymph node samples concordance, sensitivity and specificity were determined based on the comparison of these 2 methods. To investigate whether these figures might be influenced by a sampling bias caused by limited investigation of the material the histologic work-up was extended to all levels in the first 120 histologically negative lymph node samples. The same was done for paraffin blocks of discordant cases. In addition, the homogenised lymph node lysates of samples with discordant OSNA *versus* histology results were subjected to quantitative reverse-transcriptase polymerase chain reaction (QRT-PCR) and Western Blot analysis. In case these investigations yielded a result compatible with a positive OSNA result these samples were excluded from the final analysis because of a strong indication for sampling bias.

## Histological work-up

**Figure 2:**
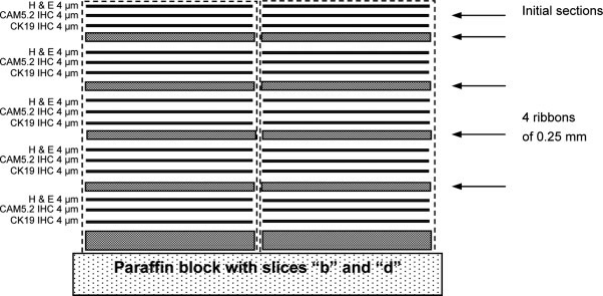
Schematic representation of the histological work-up. Both slices “b” and “d” of each lymph node sample were embedded into 1 paraffin block. Three initial 4-μm thick sections were stained with H&E, CAM5.2 and CK19, respectively. Afterwards, 4 additional levels with 250-μm skip space in between them were prepared.

Lymph nodes were cut using special cutters depending on the size. The blades of this device were 1 mm apart for lymph nodes with a minor axis of 4–6 mm and 2 mm apart for lymph nodes with a minor axis of 6–10 mm. Lymph nodes with a minor axis larger than 10 mm were halved, and the resulting pieces were then cut either with the 1 mm or 2 mm cutting device depending of the size of the pieces. Of the slices b and d initially three 4-μm thick sections were stained with H&E, CAM5.2 (Becton Dickinson, Mountain View, CA) and an anti-CK19 antibody (code No. M0888 and clone No RCK 108, Dako, Glostrup, Denmark), respectively. If the initial sections were tumor positive no further sections were cut. Otherwise, additional sections (*n* = 3) at further levels at an interval of 250 μm (usually 4) were cut and analyzed (Fig. 2). Immunostaining was performed with an antibody against cytokeratin 8 (CAM5.2) as well as CK19. Separate sections containing nonneoplastic epithelial cells were included in each staining procedure and served as a positive control for both antibodies.

The size of a metastasis was determined by measuring its largest diameter and categorized as isolated tumor cells (ITC: <0.2 mm), micrometastasis (tumor deposits larger than 0.2 mm but smaller than 2.0 mm), or macrometastasis (tumor deposits equal to or larger than 2.0 mm).^19^ Microscopic evaluation was done by 2 pathologists (MV and MJ) without prior knowledge of the results of the OSNA method. Histology was regarded positive if at least 1 micrometastasis or macrometastasis was detected in 1 of the sections. Lymph nodes containing isolated tumor cells were recorded as lymph node negative and designated as N0(i+) according to the 6th UICC TNM classification.^21^

### One step nucleic acid amplification assay for CK19 mRNA

In the OSNA method the lysate of a homogenised lymph node was directly used for amplification purposes without prior isolation and purification of RNA, as has been recently described in detail.^18^ Homogenisation was carried out with the homogenizing reagent Lynorhag, pH 3.5, (Sysmex, Kobe, Japan) and subsequent amplification with the ready-to-use Lynoamp Kit (Sysmex, Kobe, Japan) on the RD-100i (Sysmex, Kobe, Japan) according to the manufacturer's instruction. Amplification was performed by reverse transcription loop-mediated isothermal amplification (RT-LAMP) (2000),^22^ as lately presented for CK19 mRNA.^18^ Amplification products were detected by real-time monitoring of turbidity changes caused by the increase of magnesium pyrophosphate concentration, a by-product of the amplification reaction.^23^ A total of 6 primers implied a high degree of specificity for the reaction and exhibited no cross-reaction with CK19 pseudogenes a and b (Genbank accession number M33101 and U85961). The primers for the amplification of CK19 mRNA were the same as described elsewhere.^18^ The amplification process for each sample took 16 min. The processing and amplification of 3 samples took 30–40 min.

To check the quality of mRNA in the lysates amplification of the mRNA of the housekeeping gene beta-actin was performed using the following primers:

5′-TGAAGGTAGTTTCGTGGATGCCTGAGGCACTCTTCCAGC-3′ (forward inner primer), 5′-TGAAGTGTGACGTGGACATCCAGGGTACATGGTGGTGC-3′ (reverse inner primer), 5′-TGGCAATGAGCGGTTCC-3′ (forward outer primer),

5′-TCCTTCTGCATCCTGTCG-3′ (reverse outer primer),

5′-ACAGGACTCCATGCCC-3′ (loop forward primer) and 5′-TGTACGCCAACACAGTGC-3′ (loop reverse primer).

The same reagent and amplification conditions as described elsewhere^18^ were applied for both beta-actin and CK19 markers.

According to the manufacturer's specifications (Sysmex), the threshold level for the CK19 mRNA OSNA was set at 250 copies/μL lysate on the basis of previous data obtained from a series of normal lymph nodes.^18^ Consequently, in case the CK19 mRNA copy numbers, as expressed per μL of original lysate, were lower than 250 copies/μL the result was designated as (−). Copy numbers between 250 and 5,000/μL were designated as (+), and copy numbers higher than 5,000/μL as (++), as recommended by the manufacturer. In terms of comparison to results obtained by histopathology, negative (−) is consistent with negative histology or presence of ITC, scored as pN0 and N0(i+), respectively,^19^ (+) most likely is associated with a micrometastasis, and (++) with a macrometastasis as determined by basic data obtained for CK19 mRNA OSNA.^18^

### Quantitative reverse-transcriptase polymerase chain reaction

About 200 μL of the frozen lysate from discordant lymph node cases were used for extraction of total RNA with the RNeasy Mini Kit (QIAGEN, Valencia, CA). If the RNA concentration of the eluate was > 50 ng/μL, the 260–280 nm absorbance ratio (*A*_260_/*A*_280_) higher than 1.7, and the electrophoretic measurement on agarose gels yielded 2 sharp ribosomal RNA bands, QRT-PCR on the ABI Prism 7700 detector (Applied Biosystems, Foster City, CA) was performed. In case one of these criteria was not met, the RNA was considered to be of poor quality and the corresponding samples were not subjected to QRT-PCR.

Purified RNA (2 μL) was subjected to one-step QRT-PCR with QuantiTest SYBR Green (QIAGEN, Hilden, Germany) according to the manufacturer's instructions. Primers were designed by the Primer Express Version 2.0 software (Applied Biosystems). QRT-PCR was performed for CK19, SPDEF (SAM pointed domain containing ETS transcription factor), FOXA1 (forkhead box A1) and beta-actin with the QuantiTest SYBR Green (Invitrogen Corp, San Diego, CA). The latter served as house-keeping gene for an extra quality check of the RNA for RT-PCR purposes. The sequences of RT-PCR primers for beta-actin amplification were 5′-CCACACTGTGCCCATCTACG-3′ (forward) and 5′-AGGATCTTCATGAGGTAGTCAGTCAG-3′ (reverse). Primer sequences of the other markers were recently listed.^18^

All QRT-PCR reactions were performed in duplicate. Cut-off levels for CK19, SPDEF and FOXA1 were determined based on results obtained from a series of lymph nodes from breast cancer patients with and without undisputable metastases. Both cut-off level and results from the sample runs were expressed in threshold cycles (Ct). A Ct is defined as the PCR cycle in which the fluorescence exceeds a defined baseline signal. Cut-off levels for CK19, SPDEF and FOXA1 were defined as the mean Ct + 3 times the standard deviation (SD) obtained from lymph nodes of pN0 patients. The cut-off levels used were Ct 24.0 for beta-actin, Ct 31.5 for CK19, Ct 31.6 for SPDEF and Ct 33.8 for FOXA1.

### Western blot analysis

CK19 Western Blot analysis of the lysates from discordant cases was performed as previously illustrated,^18^ using A53-B/A2 (Santa Cruz Biotechnology) as the primary anti-CK19 antibody. A standard curve for CK19 protein was established based on 4 calibrators with CK19 protein concentrations of 0.15, 0.075, 0.038 and 0.018 ng/μL, respectively (Biodesign, Saco, ME). CK19 protein concentrations of the samples were identified according to this standard curve. A cut-off value for CK19 protein expression of 0.13 ng/μL (mean + 3 SDs), was determined based on the CK19 protein expression levels of 37 histologically negative lymph nodes of pN0 breast cancer patients. The results were expressed as ng/μL of original lysate.

## Results

### *OSNA-CK19* versus *histology*

In all lysates of the 346 lymph node samples beta-actin mRNA expression was detected indicating the presence of RNA of adequate quality to serve as a template for RT-LAMP.

Consequently, all 346 lymph node samples were investigated by both the OSNA assay and histology. Two hundred and sixty seven samples were negative and 61 samples positive by both methods (Table II). In 3 of these 267 tumor negative concordant cases, ITC were found by immunohistochemistry in only 1 section of the tissue part examined by histology. There were 18 discordant cases. In 15 of these samples a positive result was obtained by the OSNA-CK19 method compared to a negative histology result. On the other hand, OSNA-CK19 analysis revealed a negative result in 3 histologically positive cases. Thus the concordance rate, as determined without incorporating any additional data from discordant case investigation, between the OSNA and histological methods was 94.8%. By considering the histological procedures as the gold standard the sensitivity of OSNA for detecting tumor deposits was 95.3% and the specificity was 94.7%. To find out to what extent these figures might be influenced by a sampling bias caused by limited investigation of the material the first 120 histologically negative lymph nodes, as determined by 5 level histology, were cut into further levels at an interval of 250 μm until no remnants remained. These included 5 lymph nodes showing a positive OSNA-CK19 result. In none of these samples metastatic deposits were detected after extended analysis.

**Table II:** Comparison of the Results of OSNA-CK19 with Histological Examination

Histological work-up: 5 levels				

OSNA CK19	Macrometastases *N* = 53	Micrometastases *N* = 11	Isolated tumour cells *N* = 3	Negative *N* = 279
++	50	4	0	2
+	2	5	0	13
−	1	2	3	264

In 50 out of 53 cases with histologically detectable macrometastases, the OSNA-CK19 assay of the adjacent slice showed a (++) result, 2 cases revealed a (+) result, and 1 macrometastasis was not detected in the adjacent lymph node slice used for OSNA-CK19 (Table II). This macrometastasis (Table III, discordant case no. 1) had a diameter of 2.78 mm. 4/11 micrometastases had (++) results, 5/11 had (+) results and 2/11 revealed a (−) CK19 OSNA result. One of these micrometastases (1.5 mm in diameter, discordant case no. 2) was located in level 2 and 3 of the corresponding paraffin block but not in levels 1, 4 and 5. The other one (1 mm in diameter, discordant case no. 3) was detected only in level 2 but not in any other level.

**Table III:** Analysis of Discordant Cases by Additional Markers Indicative for Epithelial Cells

							QRT-PCR (threshold cycle)								
		
No.	Histology	OSNA		CK19 protein		RNA quality	Beta-actin^2^		CK19^3^		SPDEF^4^		FOXA1^5^		Conclusion
									
		Copies /μL	+/−	Ng/μL^1^	+/−		Ct	+/−	Ct	+/−	Ct	+/−	Ct	+/−	
1	+	ND	−	0.14	+	poor	Could not be performed								Still discordant^6^
2	+	ND	−	0.17	+	OK	19.4	+	31.8	−	32.2	−	35.4	−	Still discordant
3	+	ND	−	0.09	−	poor	Could not be performed								Still discordant
4	−	270	+	0.06	−	poor	Could not be performed								Still discordant
5	−	330	+	0.13	+	poor	Could not be performed								Sampling bias
6	−	340	+	0.04	−	OK	18.9	+	33.7	−	32.5	−	34.6	−	Still discordant
7	−	400	+	0.20	+	OK	19.1	+	33.0	−	33.0	−	34.5	−	Sampling bias
8	−	420	+	0.09	−	poor	Could not be performed								Still discordant
9	−	520	+	0.05	−	OK	19.8	+	33.0	−	33.5	−	35.7	−	Still discordant
10	−	620	+	0.05	−	poor	Could not be performed								Still discordant
11	−	640	+	0.06	−	OK	17.8	+	32.3	−	32.1	−	33.8	+	Sampling bias
12	−	710	+	0.12	−	OK	19.3	+	30.8	+	32.5	−	34.6	−	Sampling bias
13	−	720	+	0.30	+	OK	18.2	+	28.7	+	30.3	+	32.9	+	Sampling bias
14	−	730	+	0.06	−	poor	Could not be performed								Still discordant
15	−	1400	+	0.16	+	OK	20.3	+	33.6	−	32.6	−	39.0	−	Sampling bias
16	−	2500	+	3.61	+	OK	19.4	+	25.4	+	25.3	+	25.6	+	Sampling bias
17	−	12000	++	0.12	−	OK	19.1	+	33.6	−	32.4	−	38.0	−	Still discordant
18	−	12000	++	0.06	−	poor	Could not be performed								Still discordant

ND, not detected; p, poor.

In two samples (6 and 9) no CK19 protein and no CK19, SDDEF and FOXA1 mRNA was detected. In 4 samples (4, 8, 10 and 14) QRT-PCR could not be performed and Western blotting yielded negative results. Analysis of samples 11 and 12 gave only positive RT-PCR results with FOXA1 and CK19, respectively, suggestive of very small tumour deposits in these samples. In samples 5 and 7 CK19 protein concentrationabove the cut-off level was detected but no (in sample 5) or negative (sample 7) QRT-PCR results. Sample no. 13 and 16 gave positive results in Western Blot for CK19 and all 3 markers in QRT-PCR, strongly suggestive of the presence of tumor cells in the lysate. A positive OSNA-CK19 result with high copy number was observed in 2 samples (17 and 18), with no metastases detected by histological methods. In sample 15, CK19protein was present. In samples 17 and 18 data obtained by Western blotting and RT-PCR were negative or could not be performed, respectively.

1 Cut-off level: 0.13 ng/μL, cut-off value as indicated in threshold cycles (Ct).

2 Beta-actin: 24.0.

3 CK19: 31.5.

4 SPDEF: 31.6.

5 FOXA1: 33.8.

6 Still discordant: 8 of these cases could not be investigated by RT-PCR due to poor RNA quality.

### Discordant case investigation

To investigate if discordant results between OSNA-CK19 and histological examination resulted from a sampling bias such that tumor deposits were found exclusively in either slices used for histology, b&d, or slices used for OSNA, a&c, the homogenate of “a” and “c” slices of all these samples was further investigated by QRT-PCR for mRNA markers indicative for epithelial cells (CK19, SFDEF and FOXA1) as well as for CK19 protein by Western Blotting, whenever possible (Table III). Moreover, in addition to the 5 samples belonging to the first 120 histologically negative samples subjected to extended analysis, also paraffin blocks b&d from the 10 remaining OSNA positive, histology negative samples were further serially sectioned until no remnants remained, and these sections were stained by H&E, CAM 5.2 and an anti-CK19 antibody. No tumor deposits were detected with these additional histological analyses.

In 8 cases, only Western Blot analysis for CK19 protein could be obtained because poor quality RNA did not allow QRT-PCR (Table III). One (no. 2) of 3 histology positive/OSNA-CK19 negative samples (no. 1–3) could be analyzed by QRT-PCR and yielded negative results for all 3 markers. In the lysates of samples nos. 1 and 2, CK19 protein levels of 0.14 ng/μL and 0.17 ng/μL, respectively, were slightly above the cut-off level suggesting the presence of small tumor deposits. In 11 histologically negative samples (no. 4–14) low CK19 mRNA copy numbers (250–750/μL) were found with OSNA-CK19. Six of these could be further analyzed by QRT-PCR,^6^,^7^,^9^,^11^–^13^ whereas the remaining 5 samples suffered from poor RNA quality. The same was true for 1 sample (No. 18) with high CK19 mRNA copy number (Table III).

In summary, in 7 out of 18 discordant samples (no. 5, 7, 11, 12, 13, 15 and 16) sampling bias was suggested to be the reason for discordant data since at least 1 of the additional analyses results was compatible with a positive OSNA-CK19 result (Table III). If these 7 samples were excluded from the study because of suspicion for sample allocation bias, sensitivity remained 95.3% (61/64) but specificity and concordance rate increased to 97.1% (267/275) and 96.8% (328/339), respectively.

## Discussion

Since there is no fast method for high quality lymph node assessment available, current histology-based SN protocols require a second surgical procedure once the node is found to be tumor positive.^24^

In the present study a novel OSNA-CK19 method was compared to immunohistological examination at 5 levels on 346 ALNs of 32 breast cancer patients. OSNA-CK19 had a sensitivity of 95.3% and a concordance rate of 94.8%, when the results of further discordant case analyses were not taken into consideration. Of the 3 histologically positive samples that were negative in the OSNA-CK19 assay, 2 were attributed to micrometastases and 1 to a small macrometastasis with a diameter of 2.78 mm (Table III). Without considering the results of discordant case investigation the specificity of OSNA was 94.7%. For 7 of the 18 discordant cases a sampling bias was likely to be the case on the basis of further QRT-PCR and Western Blot data. If these samples were excluded from the analysis the specificity and concordance rates increased to 97.1 and 96.8%, respectively, without affecting the sensitivity. Of 8 additional discordant cases that could not be analysed by QRT-PCR tissue allocation bias cannot be excluded and therefore these cases are not necessarily false-positive in OSNA. The reason that the RNA in the lysates of other discordant samples was of insufficient quality for further analysis most likely results from the fact that the lymph node lysates were stored after the OSNA run for a couple of months before discordant case investigation was performed. In addition, the homogenisation buffer used for the OSNA method is not the optimized homogenising solution recommended by QIAGEN as part of their RNA purification protocol.

It should be noted that both slices a&c were completely analyzed by OSNA, whereas the 250-μm skip ribbons of slices b&d used for histology were discarded. In this way more tissue is used for the OSNA investigation and therefore a higher sensitivity of the OSNA assay for tumor deposits as compared to histologic work-up can be expected. The high sensitivity and specificity and the short analysis time make OSNA-CK19 the method of choice for rapid assessment of lymph node metastases in breast cancer patients.

It should be noted that isolated tumor cells (≤0.2 mm) are not detected by OSNA-CK19 and are classified as lymph node negative. Presently, lymph nodes containing ITC, as demonstrated by histology are clinically considered as metastasis negative and designated as N0(i+) by TNM classification.^19^ Most ITC are found by immunohistochemical staining only and not by H&E staining.^25^ At the moment the clinical significance of ITC in lymph nodes of breast cancer patients is unknown, and currently there is no consensus regarding ALND or further treatment in the presence of ITC.^25^,^26^

Several earlier studies described CK19 mRNA expression in lymph nodes from patients without cancer, producing false positive results.^27^–^29^ To prevent this, a CK19 mRNA cut-off level of 250 copies/μL lysate, as determined in a different study,^18^ was used in OSNA-CK19. By using this approach any illegitimate CK19 mRNA background expression sometimes present in lymph nodes will fall below the cut-off level of the method and will not interfere with the detection of tumor deposits. Moreover, 6 primers were chosen in such a way that pseudogenes are not amplified. In conclusion, the OSNA method based on CK19 mRNA as described in our study is an attractive intra-operative tool for the detection of lymph node metastases in breast cancer patients. Since the whole lymph node can be investigated, sampling errors, which are inherent to histological techniques, are prevented by OSNA. Alternatively, a small part of the lymph node could be reserved for other investigations. However, the larger the part of the lymph node that is excluded from the OSNA analysis the higher the chance that tumor deposits might be missed. Due to an automated pipetting procedure and ready-to-use reagent kit a high degree of standardization and objectivity is achieved. Intra-operative use of OSNA may spare a patient from the discomfort and complications of a second surgical intervention. Additional studies including intra-operative OSNA analyses of sentinel lymph nodes are currently underway.

## Abbreviations

ALND: axillary lymph node dissection
CK19: cytokeratin 19
Ct: threshold cycle
FOXA1: forkhead box A1
H&E: Hematoxylin and Eosin
ITC: isolated tumor cells
OSNA: one step nucleic acid amplification
QRT-PCR: quantitative reverse-transcriptase polymerase chain reaction
RT-LAMP: reverse transcription loop-mediated isothermal amplification
SD: standard deviation
SN: sentinel node
SPDEF: SAM pointed domain containing ETS transcription factor.

## References

[1] Kim T, Giuliano AE, Lyman GH (2006). Lymphatic mapping and sentinel lymph node biopsy in early-stage breast carcinoma. Cancer.

[2] Wilson LL, Giuliano AE (2005). Sentinel lymph node mapping for primary breast cancer. Curr Oncol Rep.

[3] Ferrari A, Rovera F, Dionigi P, Limonta G, Marelli M, Besana Ciani I, Bianchi V, Vanoli C, Dionigi R (2006). Sentinel lymph node biopsy as the new standard of care in the surgical treatment for breast cancer. Expert Rev Anticancer Ther.

[4] Mabry H, Giuliano AE (2007). Sentinel node mapping for breast cancer: progress to date and prospects for the future. Surg Oncol Clin N Am.

[5] Brogi E, Torres-Matundan E, Tan LK, Cody HS (2005). The results of frozen section, touch preparation, and cytological smear are comparable for intraoperative examination of sentinel lymph nodes: a study in 133 breast cancer patients. Ann Surg Oncol.

[6] Dowlatshahi K, Fan M, Anderson JM, Bloom KJ (2001). Occult metastases in sentinel nodes of 200 patients with operable breast cancer. Ann Surg Oncol.

[7] Tanis PJ, Boom RP, Koops HS, Faneyte IF, Peterse JL, Nieweg OE, Rutgers EJ, Tiebosch AT, Kroon BB (2001). Frozen section investigation of the sentinel node in malignant melanoma and breast cancer. Ann Surg Oncol.

[8] Van Diest PJ, Torrenga H, Borgstein PJ, Pijpers R, Bleichrodt RP, Rahusen FD, Meijer S (1999). Reliability of intra-operative frozen section and imprint cytological investigation of sentinel lymph nodes in breast cancer. Histopathology.

[9] Torrenga H, Rahusen FD, Meijer S, Borgstein PJ, van Diest PJ (2001). Sentinel node investigation in breast cancer: detailed analysis of the yield from step sectioning and immunohistochemistry. J Clin. Pathol.

[10] Van Diest PJ, Torrenga H, Meijer S, Meijer CJ (2001). Pathologic analysis of sentinel lymph nodes. Semin Surg Oncol.

[11] Cserni G, Amendoeira I, Apostolikas N, Bellocq JP, Bianchi S, Bussolati G, Boecker W, Borisch B, Connolly CE, Decker T, Dervan P, Drijkoningen M (2003). Pathological work-up of sentinel lymph nodes in breast cancer. Review of current data to be considered for the formulation of guidelines. Eur J Cancer.

[12] Weigelt B, Verduijn P, Bosma A, Rutgers EJ, Peterse HL, Van't Veer LJ (2004). Detection of metastases in sentinel lymph nodes of breast cancer patients by multiple mRNA markers. Br J Cancer.

[13] Gillander WE, Mikhitarian K, Hebert R, Mauldin P, Palesch Y, Walters C, Urist M (2004). Molecular detection of micrometastatic breast cancer in histopathology-negative axillary lymph nodes correlates with traditional predictors of prognosis: an interim analysis of a prospective multi-institutional cohort study. Ann Surg.

[14] Abdul-Rasool S, Kidson SH, Panieri E, Dent D, Pillay K, Hanekom GS (2006). An evaluation of molecular markers for improved detection of breast cancer metastases in sentinel nodes. J Clin Pathol.

[15] Chu PG, Weiss LM (2002). Keratin expression in human tissues and neoplasms. Histopathology.

[16] Horibe D, Ochiai T, Shimada H, Tomonaga T, Nomura F, Gun M, Tanizawa T, Hayashi H (2006). Rapid detection of metastasis of gastric cancer using reverse transcription loop-mediated isothermal amplification. Int J Cancer.

[17] Taniyama K, Motoshita J, Sakane J, Makita K, Akai Y, Daito M, Otomo Y, Ono H, Mizunoe T, Takeuchi Y, Tominaga H, Koseki M (2006). Combination analysis of a whole lymph node by one-step nucleic acid amplification and histology for intraoperative detection of micrometastasis. Pathobiology.

[18] Tsujimoto M, Nakabayashi K, Yoshidome K, Kanedo T, Iwase T, Akiyama F, Kato Y, Tsuda H, Ueda S, Sato K, Tamaki Y, Noguchi S (2007). One-step nucleic acid amplification (OSNA) for intraoperative detection of lymph node metastasis in breast cancer patients. Clin Can Res.

[19] Singletary SA, Greene FL, Breast Task Force (2003). Revision of breast cancer staging: The 6th edition of the TNM classification. Semin Surg Oncol.

[20] Giettema HA, Vuylsteke RJCLM, De Jonge IA, Van Leeuwen PAM, Molenkamp BG, Van der Sijp JRM, Meijer S, Van Diest PJ (2004). Sentinel lymph node investigation in melanoma: detailed analysis of the yield from step sectioning and immunohistochemistry. J Clin Pathol.

[21] Sobin LH, Wittekind C (2002). UICC: TNM classification of malignant tumours.

[22] Notomi T, Okayama H, Masubuchi H, Yonekawa T, Watanabe K, Amino N, Hase T (2000). Loop-mediated isothermal amplification of DNA. Nucleic Acids Res.

[23] Mori Y, Nagamine K, Tomita N, Notomi T (2001). Detection of loop-mediated isothermal amplification reaction by turbidity derived from magnesium pyrophosphate formation. Biochem Biophys Res Commun.

[24] Cserni G, Bianchi S, Boecker W, Decker T, Lacerda M, Rank F, Wells C (2005). Improving the reproducibility of diagnosing micrometastases and isolated tumor cells. Cancer.

[25] Lannin D (2004). How many breast cancer cells in a sentinel lymph node are OK. Ann Surg Oncol.

[26] Lyman GH, Giuliano AE, Somerfield MR, Benson AB, Bodurka DC, Burstein HJ, Cochran AJ, Cody HS, Edge SB, Galper S, Hayman JA, Kim TY (2005). American Society of Clinical Oncology guideline recommendations for sentinel lymph node biopsy in early-stage breast cancer. J Clin Oncol.

[27] Yun K, Gunn J, Merrie AEH, Phillips LV, McCall JL (1997). Keratin 19 mRNA is detectable by RT-PCR in lymph nodes of patients without breast cancer. Br J Cancer.

[28] Bostick PJ, Chatterjee S, Chi DD, Huynh KT, Giuliano AE, Cote R, Hoon D (1998). Limitations of specific reverse-transcriptase polymerase chain reaction markers in the detection of metastases in the lymph nodes and blood of breast cancer patients. J Clin Oncol.

[29] Merrie AEH, Yun K, Gunn J, Philipps LV, McCall JL (1999). Analysis of potential markers for detection of submicroscopic lymph node metastases in breast cancer. Br J Cancer.

